# Reproductive health among Venezuelan migrant women at the north western border of Brazil: A qualitative study

**DOI:** 10.1016/j.jmh.2021.100060

**Published:** 2021-07-07

**Authors:** Maria Y. Makuch, Maria Jose D. Osis, Cinthia Brasil, Helder S.F. de Amorim, Luis Bahamondes

**Affiliations:** a Department of Obstetrics and Gynaecology, Faculty of Medical Sciences, University of Campinas (UNICAMP), Caixa Postal 6181, Campinas 13084-971, SP, Brazil; b Centre for Reproductive Health in Campinas (Cemicamp), Campinas, SP, Brazil; c Department of Collective Health, Faculty of Medicine of Jundiaí, Jundiai, SP, Brazil; d Direction of Basic Attention Care, Health Secretary, Municipality of Boa Vista, Boa Vista, RR, Brazil; e Direction of Basic Attention Care, Health Secretary, State of Roraima, Boa Vista, RR, Brazil

**Keywords:** Migrants, Women, Venezuela, Brazil, Sexual and reproductive health, Focus groups, Qualitative study

## Abstract

**Background:**

Venezuela has been immersed in an economic and social crisis with a high number of migrant people. An important proportion of Venezuelan migrants have crossed the north western border Brazil-Venezuela were the United Nations High Commissioner for Refugees (UNHCR) has established 13 shelters. Our objectives were to know perspectives and views of Venezuelan migrant women hosted at UNHCR shelters on some SRH issues.

**Methods:**

We conducted a qualitative study between November 2019 and February 2020 with 12 focus group discussions (FGDs), with 111 Venezuelan migrant women of reproductive age (18–49 years old). FGDs were performed in a closed space that guaranteed confidentiality, were recorded, verbatim transcribed and data were analised for thematic manifest content.

**Findings:**

The themes identified were perspectives on: i) health care for pregnant and postnatal women, ii) access to modern contraceptive methods, and iii) HIV and sexually transmitted diseases (STDs). Despite the general satisfaction with obstetric care, women noted few challenges pertaining to their experiences during first entry to antenatal care, labour, delivery and postnatal care. They were in agreement that access to long-acting reversible contraceptives was difficult, mainly to the copper-intrauterine device (IUD); which when available it was erratic. Hormonal-IUD and implants were almost inexistent. This was of major concern to the women, as it prevented them from the ability to plan their reproductive lives. Although knowledge on STDs/HIV prevention and transmission was adequate; the predominance of traditional gender imbalance in the relations was observed and these attitudes have been discussed as a barrier for migrant women to protect themselves against HIV/STD infection.

**Conclusion:**

These migrant women needed help to understand the language and functioning of the healthcare system, to overcome barriers and challenges while seeking access to SRH care. They faced significant gender vulnerability that needs to be addressed within their new life. Our findings could be useful for health authorities and international organisations to start actions to improve SRH and mitigate suffering.

## Introduction

It is estimated that there are almost 71 million displaced people worldwide, and almost half of them are adolescent girls and women of reproductive age (Askew et al., 2016; UNHCR). Women are particular vulnerable because of the risk of poor sexual and reproductive health and rights (SRHR) outcomes and because in many cases they are victims of sexual and domestic violence (Finnerty and Shahmanesh, 2017). An important proportion of these women need to use more often SRH services for pregnancy, childbirth and eventual complications, safe and unsafe abortions, contraceptive provision including placement of intrauterine devices (IUDs) and implants, gynaecological complaints, gender-based violence, among others (Sami et al., 2014; Inter-Agency Working Group 2017; Albaladejo, 2018).

During the last years, Venezuela is immersed in an economic and social crisis (UNHCR). The health system is also immersed in an emergency and many health indicators were worsened, including those regarding SRHR (World Health Organization; UNICEF; UNHCR 2012) an important public health issue worldwide (Askew et al., 2016), and particularly important for migrants and refugee women, living in settings in which the healthcare system receives increased demands for assistance for which generally the systems are not well prepared (Askew et al., 2016; Inter-Agency Working Group 2017; World Health Organization; UNICEF; UNHCR 2012; UN Agency). In Venezuela the maternal mortality ratio, adolescent pregnancy and shortages of contraceptive methods reported were higher when compared to the other Latin American countries (Albaladejo, 2018; UNHCR; UN Agency; 10; Page et al., 2019; Doocy et al., 2019).

The number of people fleeing the country has increased dramatically in the most recent years and the International Organisation for Migration (IOM), in 2019, has estimated that more than 4.5 million Venezuelans had fled the country, and the United Nations High Commissioner for Refugees (UNHCR) has reported that 650,000 Venezuelan asylum applications in 2018 and about 2.1 million are living under other legal forms of stay in the Americas (UN Agency).

Brazil has also received a significant number of Venezuelan migrants and it is estimated that more than 289,000 Venezuelans arrived and stayed in Brazil up to December 2019 (10; Page et al., 2019) and hosts the sixth largest overall migrant population in South America (Page et al., 2019; Doocy et al., 2019). Most of the Venezuelan migrants enter Brazil through the north-western border at the state of Roraima, and it is estimated that 40,000 Venezuelans are living in the two main cities of the state; Pacaraima, a city at the border, and Boa Vista, the state capital, representing at the end of the year 2019 about 10% of the local population. To attend the demand of the migrants, in Brazil the UNHCR in collaboration with the Brazilian Army have established 13 shelters and the country give access to healthcare system at no cost. These conditions offered to migrants differ from those offered by Colombia, Ecuador and Peru, were usually shelters have not been settled because the authorities have privileged the transportation of migrants to other places (13; 14; Rivillas-García et al., 2021).

There is scarce and fragmented data on the needs of Venezuelan migrant women regarding SRHR and on their perspective on the access and barriers to receive SRHR services (Page et al., 2019; Doocy et al., 2019). Our objectives were to study perspectives and views of Venezuelan migrant women of reproductive age sheltered in Roraima State, Brazil on SRH and the health care they received following Inter-Agency Working Group (IAWG) on Reproductive Health standards tools (Inter-Agency Working Group 2017).

## Materials and methods

### Study design and participants

This qualitative phenomenological descriptive study (Willis et al., 2016) was part of a mixed methods study conducted between November 2019 and February 2020 at the north-western border of Brazil-Venezuela with Venezuelan migrant women of reproductive age. The study design was adapted from the Minimum Initial Service Package (MISP) readiness assessment tools from the IAWG on Reproductive Health (Inter-Agency Working Group, 2017). Our research protocol was approved by the Ethics Committee of the University of Campinas, Campinas, SP, Brazil. The quantitative data was published previously (Bahamondes et al., 2020) and in this report we analysed qualitative data regarding SRH excluding gender-based violence which will be reported separately.

Our study allowed us to understand the impact of this crisis from women's perspectives, by shedding light on their emotions, beliefs and values, actions and behaviors; and to understand women's responses to SRH related experiences, and the meanings these experience has for them. We conducted focus group discussions (FGDs) to obtaining individual and shared group views on the SRH issues (Krueger and Casey, 2000; O'Brien et al., 2014; Braun and Clarke, 2006).

Participants of the FGDs were migrant women between 18 and 49 years old, hosted at five UNHCR shelters based in Boa Vista, Roraima, Brazil located 200 km from the main land crossing points from Venezuela. Shelters were selected according to the logic of purposive sampling, aiming to cover the shelters that hosted the largest number of migrant women, according to information provided by the UNHCR. The women who participated in the FGDs were hosted at these shelters, within the age range, and voluntary signed an informed consent prior to their inclusion in the study. Variation in age and length of time of living in the shelters were considered in order to obtain diversity among the experiences and views of the participants. The number of FGDs performed was previously determined as two FGDs by selected shelter. At the end of each field work day both researchers discussed the data obtain in each FGDs and a brief resume was drafted. Based on that information data saturation was confirmed through consensual agreement that the overall data was meaningful for the objectives proposed for the study and that no new information was emerging from the FGDs (Krueger and Casey, 2000; O'Brien et al., 2014; Braun and Clarke, 2006). We followed throughout this study the Standards for Reporting Qualitative Research guidelines (Braun and Clarke, 2006).

### Procedures

**Fig. 1:**
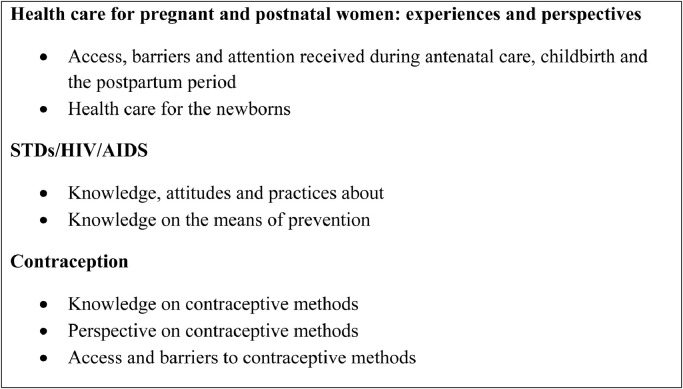
Main topics on SRH included in the FGDs guide. SRH: Sexual and Reproductive Health; FGD: Focus groups discussions, STD: Sexual transmitted diseases.

Focus groups were organised at a convenient time for the women and did not interfere with shelter activities. In all the selected shelters women were firstly informed about the FGDs by members of the agencies working at the site, subsequently the researchers of the FGDs explained the objectives of the research, how FGDs would be conducted and invited women to participate. All the FGDs were performed in a closed space that guaranteed privacy and confidentiality and allowed women to express themselves freely. There was no financial compensation for the participants. FGDs were organised with 4 to 12 women, with the exception of two groups that had 14 women each. All FGDs were conducted using a semi-structured guide and initiated with a broad open-ended question “*Please tell me about your experience of living in a shelter*”; followed by more specific questions on SRH issues (Fig. 1). Open-ended questions and probes were used to ensure feedback from the participants and facilitate a more in-depth discussion.

All the FGDs were conducted by two social scientists, a psychologist and a sociologist (MYM and MJDO) with large experience in qualitative studies and SRH issues. The FGDs were recorded digitally and ranged between 60 and 100 min. Confidentiality was maintained by identifying FGD and participants with a number. All audiotaped data and transcripts were saved in a password protected computer. Prior to the initiation of each FGD a form with some socio-demographic characteristics of each participant (age, ethnicity, cohabitation status, number and age of children, schooling, type of job before migrating, time living in the shelter and paid work at the time of the FGDs) were filled out by each participant.

### Data analysis

We used an inductive approach to perform a conventional thematic analysis of the manifest content (Braun and Clarke, 2006). FGDs audio recording were professionally transcribed, following which the transcripts were checked by two researchers (MYM and MJDO) against the recordings to ensure accuracy and completeness. During the initial phase of analysis, in the process of reading through the interviews and compiling salient topics, the recurring ideas, experiences and behavioural patterns were organised searching for and generating meaningful themes responding to the research question. Mayor themes were identified and initially coding was performed manually. This process of coding was done independently by two of the authors (MYM; MJDO) and reviewed by another author (LB). Themes and sub-themes were compared, discrepancies were discussed by all authors ensuring that results were presented and organised following a conscious and transparent process of describing the emerging data referred to SRH experiences of the women.

Quotes were also selected following the strategy of consensus among the authors.

Subsequently NVivo Qualitative Data Analysis Software (Version 12, 2020, QSR International Pty Ltd.) was used for continued data management, coding process and analysis. All FGDs were conducted in Spanish and quotations were translated by one author (MYM) into English and back to Spanish by another author (LB) to assure that the meaning was not changed during translation. The main themes identified were organised in the following categories of analysis: i) perspective on health care for pregnant and postnatal women, ii) perspective on the access to contraceptive methods and iii) perspective and knowledge regarding HIV and other sexually transmitted diseases (STDs). Each excerpt includes the number of the FGD for “Focus Group Discussions”.

## Results

Twelve FGDs were carried out with 111 Venezuelan migrant women living in the selected shelters. Table 1 shows some characteristics of these women. The age (mean ± SD) was 34.4 ± 11.8 years old and the length of time living at the shelter was 5.5 ± 4.9 months. According to the participants, women, men and children living in the shelters, in general, had difficulties of access to public health services. Many referred to problems of discrimination in the public health network and considered that one of the reasons was that they were not fluent in Portuguese, which posed a barrier to establish communication with healthcare providers and in their view constituted one of the reasons for not receiving adequate medical attention. For other women the main reason was the fact that many Brazilians considered that there were too many Venezuelan migrants with health problems and that these migrants took their place in the healthcare system creating long waiting lines to receive health care.

**Table 1:** Characteristics of women who participated in the FGDs (*n* = 111).

Age (Mean ± SD) (years)	34.4 (± 11.8)
Children with the migrant woman (Mean± SD)	2.5 (± 1.1)
Time living in the shelter (months) (Mean± SD)	5.5 (± 4.9)
***Ethinicity (n*** ***=*** ***108)***	N (%)
White	25 (23.1)
Black	16 (14.8)
Biracial	60 (55.6)
Asian	7 (6.5)
***Cohabitation status (n*** ***=*** ***104)***	
With a partner	63 (60.6)
Without a partner	41 (39.4)
***Migrated with (n*** ***=*** ***109)***	
Children and a partner	39 (35.7)
Only with children	35(32.1)
Only with a partner	4 (3.6)
Children and other relatives	13(11.9)
Alone	9 (8.2)
Only with other relatives	9 (8.2)
**Educational level (*n*** **=** **110)**	
Illiterate	2 (1.8)
Primary	16 (14.5)
High school	61 (55.4)
Post-secondary school	31 (28.3)

### Perspective on health care for pregnant and postnatal women

Most women acknowledged that there were scarce problems in the access to antenatal care (ANC). Some considered that eventually it was difficult to schedule the first ANC consultation; however, they also recognised that after performing the first consultation there was no problem to continue ANC controls that were scheduled according to the Brazilian national rules.

“ … when you go the first time to ask for a consultation … you know, your antenatal visit, afterwards they schedule your other visits. They say come this day and you go that day and your visit is scheduled.” (FGD 10).

There was agreement that the overall medical attention pregnant women received at the Maternity hospital was very good, both during ANC and hospitalisation for childbirth; women were well treated by all healthcare professionals, and were carefully examined and received the medicines they needed.

*“My daughter had her baby here …when we went to the maternity, I tell you the attention could not have been better, it was excellent. I thank God that with my daughter everything went well.”* (FGD 2)

“*Then they gave her diapers for the baby and protection for her, sanitary pads for her … so I say that, in my opinion, the care my daughter received was excellent*.” (FGD 2)

Some participants discussed the issue that there were some pregnant women living in the shelters that only received one ANC consultation because they had difficulties of access to the first consultation at the Maternity hospital. However, this situation became less frequent when pregnant women started to have access to ANC at the primary health posts. A benefit for pregnant women, albeit not always available, was the payment for the transportation to the facility in which they received ANC, considered important when the consultation was far away from the shelter. On occasions, that were scarce, when this benefit was not available pregnant women had to resolve the situation on their own.

“*They facilitate an ambulance or a taxi for women in labour … It depends if the health service is far away or close. The health post of this area is close so we can easily walk*.” (FGD 6)

Participants discussed the difficulties that pregnant women had to schedule the routine antenatal tests and exams because the waiting time for an appointment sometimes was long and this was an important reason for some women not completing all the antenatal routine. Another relevant reason discussed was that exams, such as ultrasound scan or specific exams for high risk pregnancies were performed at different facilities, sometimes located far away from the shelters and the transportation provided by the humanitarian organisations was not always available.

“*To do antenatal exams is difficult. I am 5 month pregnant and I have not been able to do the exams because there are no schedules available.”* (FGD 12)

When women were in labour transportation was provided to take them to the Maternity hospital. The reasons discussed for the lack of transportation for women in labour were that there was one ambulance, and that it had occurred that it was taking another patient to a hospital when called for a woman in labour. The other situation discussed was that, at times, there were no resources to call a taxi.

*“Women have to be having the baby for the transportation to take them. One of them had the baby in the ambulance on the way to the hospital.”* (FGD 6)

Participants said that most of the women at the shelters considered that pain during labour was normal; however, some referred to the fact that it was much commented that during childbirth women “suffered” because at the Maternity hospital healthcare providers were more in favour of vaginal delivery rather than Caesarean delivery. Moreover, the participants reported differences in intrapartum care practices between Venezuela and Brazil. The main difference was the fact that at the Maternity hospital, Caesarean delivery was only decided by the Obstetrician as a medical indication, and not as a mean to alleviate suffering during labour. Another situation that was much discussed and considered different from Venezuela was the fact that nurses monitored labour and that the physician was only called when the nurse considered it was necessary. It was also reported that women in labour and during childbirth were allowed to have one person of their choice with them and that companion was allowed to be there all the time. This social-medical approach was considered very different to what they were expecting, because in Venezuela women were not allowed to have a companion.

*“…they leave you alone during labour …. Because there are many women labour and the doctors cannot wait with each one to have the baby, because there are so many deliveries, 300 I believe, I don't remember exactly.”* (FGD 12)

*“That is what I have heard in many opportunities … and it's like that, a woman that gave birth told me that the companion the women choose can stay in the room with her and when they [companion] see that the baby is being born, that the baby's head is low the companion calls the doctor and the doctor comes and attends the birth of the baby*.” (FGD 12)

In some FGDs participants said that there are women who are afraid to go out of the shelter for childbirth in an institution they do not know and in a different environment. However, it was reported that only a few births occurred in the shelters. When that was the situation other women who already have children or migrant midwife or nurse living in the shelter have helped the labouring women. In general, women considered that medical care in the postnatal period was good and that they were well taken care off and only received discharge from the Maternity hospital when they had fully recovered and when it was verified that the baby had no problems. After hospital discharge postnatal controls continued at a primary health post close to the shelter.

“*One thing that I liked in the care after the baby was born was that until they were sure that the baby was in perfect health they do not discharge you, and that in my opinion is very good*.” (FGD 4)

Some women discussed the fact that there were many pregnant women at the shelters, and considered that some women became pregnant because they knew that if they have a child born in Brazil they obtain automatically the legal residence for them and their family. The other reason discussed was that some women when they become pregnant in Venezuela they migrate to Brazil because in their country obstetric attention was very deficient, there are no supplies at the maternity hospitals and they have to pay for all the supplies necessary for delivery.

*“I heard, the following explanation: because I want my child to have the Brazilian nationality; that is one of the explanations, and the other one: I will give birth here so my baby is Brazilian and they will give me the Brazilian nationality… that is what I have heard.”* (FGD 7)

*“In Venezuela to have a baby, when you go to the hospital, you have to take all the things that you need to use during delivery, gloves, gaze.”* (FGD 10)

Another reason to become pregnant discussed during FGD was the hope these women had to improve their life conditions by migrating to Brazil and the possibility to provide their children with the necessary things for their development, a situation they did not consider possible in their country.

*“Those who do not have children want to have children here, because they have the opportunity to give their child what they can't give them in Venezuela.”* (FGD 7)

### Perspective on the access to contraceptive methods

In general, it was reported in all the FGDs that in 2019 there were no or scarce difficulties to access combined oral contraceptives (COC), injectables and condom, different than IUD and implants which were almost inexistent. Participants said that male and female condom, were available on demand and that male condoms were also available at the shelters. It was also mentioned in one of the FGDs that to obtain COC or injectables women needed a previous consultation with a gynaecologist.

*“… but it has to be a gynaecologist, you can't go and ask for it (pill) … you cannot ask for it at the reception. Once I had a consultation with a cardiologist and he did not give me the referral he said I had to see a gynaecologist.*” (FGD 4).

Women acknowledged that injectable contraceptive were much required by the migrants, and this method, in general, had been available at the health facilities until the end of the previous year. However, at the time when the FGDs were conducted, (January 2020), in some FGDs there was agreement on the lack over the last few months of COC, Cu-IUD and injectables.

*“Now it is … it is something complicated … injectables are not available at the health post … you cannot get the injectable in all the health posts and also you cannot get the copper T and the chip (implants) … I did not get them.*” (FGD 7)

*“Yes, I have heard some comments, once there was a woman who got a referral to get the 3 month injectable. You go to the health post, they give you the referral to go and get the injectable, then you come back here (to the shelter) and they give you the injection here.*” (FGD 2)

In some FGDs the women commented that it was possible to have access to Cu-IUD and implants. However, when they discussed availability of these contraceptives at the health facilities, they affirmed that those methods were no available. Additionally, some women said that the Cu-IUD was available at the Women referral centre and Maternity hospital, and others added that at that facility it was also possible to obtain contraceptive implants. It was also explained that women who requested Cu-IUD insertion needed a referral from the primary health post or after delivery. In the case of a postpartum period, women had to wait six weeks after childbirth before IUD insertion. In one FGD the possibility of immediate postpartum Cu-IUD placement was discussed. However, at the time that FGDs were conducted the Cu-IUD was not available.

*“Yes, I got it [copper T] in the referral centre for woman's health … they are giving it to many women.”* (FGD 9)

*“When my baby was born I asked for the apparel and they told they did*

*not have them. I went to the health post and asked for a referral to centre for woman's health, and they don't have.”* (FGD 7)

Almost all women agreed that there is difficult access to tubal ligation. It was considered a good contraceptive method for migrant women who live in shelters who did not want to have more children and who were in the situation of starting a new life in a new country. One of the women referred to her personal experience, that tubal ligation, had been refused and she was counselled to use a Cu-IUD.

*“It is very difficult to have a referral for (tubal) ligation… you have to assist an*

*educational activity and perform a series of exams.* (FGD 12)

*“Now we do not want many children, because we also think in a future in*

*Venezuela …. They should help us. Venezuelan women don't want to have more children.”.* (FGD 12)

In one of the FGDs women discussed that one of the difficulties women had to go to the healthcare facilities to obtain contraceptive methods was that they had children and could not leave them without attention at the shelter.

*“It is also difficult for many women here, they cannot go out (of the shelter)*

*because they have two, tree, four children, and they don't have anybody to take care of their children because they are women that are here alone, they go to the health facility only when some friend can take care of their children.*” (FGD 11)

### Perspective and knowledge regarding HIV and other STDs

There was a general agreement that HIV/AIDS is a STD and that it was important and necessary to prevent those diseases. The women also said that they have heard or knew there were people living with HIV or AIDS in the shelters. However, they never received any information regarding this situation.

*“There were rumours here about cases of HIV, that at the shelter X there were*

*200 confirmed cases, I don't know if it's true or if it's a rumour because sometimes I don´t know if it is true … but here, some time ago, there was a confirmed case of HIV, then there were many educational activities about HIV, there were people who did not know much about this disease and at that time much information was transmitted.*” (FGD 7)

*“Not long ago, a few months ago, here in the shelter a woman died of HIV … a*

*few times I was with her, close to her and I even hugged her.”* (FGD 8)

In one of the FGDs women discussed that there had been a woman with HIV in their shelter that had been transferred to a hospital in São Paulo for treatment. According to the participants this woman had said that the treatment she had received since she arrived in Brazil had been very good.

*“I know a woman, now she is in São Paulo, she was infected … she said she had*

*a very good impression of the services here were she was treated because she was priority because of her sickness.”* (FGD 4)

In one of the FGDs women had some doubts on the ways of transmission of HIV and some mentioned that they had heard that contamination can occur using the same glass that a contaminated or sick person has used.

*“But there are people who say: if I drink of this glass and that person also*

*drinks then you can get it (HIV).”* (FGD 9)

“When I arrived at the shelter there was one case, a young woman that was positive for HIV, and there were many rumours. The people from the organisation of the shelter took action because the residents became very uneasy with this issue, because this woman did not have her own bathroom and she used the female bathroom, she urinated there … and many times she was bleeding so the women were alert with that.” (FGD 9)

The women said that they knew other STDs such as syphilis, HPV, candidiasis, cancroid and gonorrhoea. They also mentioned “*unspecific vaginal infections*”. It was also common that women during FGDs associated problems of hygiene and inefficient cleaning routines of the bathrooms of the shelters with an increased risk for women and children to acquire a STD.

“At the same time there was an increase of many people with different sexually transmitted diseases, and they are there and they don't say anything and they share the sanitary facilities with us.” (FGD 9)

“I believe this is a delicate issue, if the people from the organisation of the shelter know that these people with this kind of disease are at the shelter, they should take action to maintain them separated, not because of discrimination, but because it is an action that means safety for us and for the children.” (FGD 9)

When asked about what the means of prevention for STDs were, in general, discussions focussed on male condoms use and monogamy. Most of the women considered that women could not have confidence in their partners, being or not in a stable relationship. Their perspective was that multiple partners and conjugal infidelity are “normal” for men and that was the attitude women expected from men. Considering this situation, they said that women should make it clear to men that they should use condom in extra conjugal relationships so they will not transmit STDs to their stable partner. Nevertheless, in some FGDs there were women who considered that prevention of STDs was based on a stable, honest and monogamous relationship. In general, when this perspective was discussed many participants disagreed and considered that it was a naïve and unrealistic perspective.

*“Then I always tell him (husband) there is HIV here, there is HIV around the*

corner… I have stopped breastfeeding my baby, and I always told him (husband) remember that if I get sick the baby will be sick also, and if the baby is sick I will also get sick … then you have to think about these things, because men are men, it's there nature not to be faithful, so remember there are condoms …” (FGD 6)

*“One can have a stable partner but you don't know if that partner is really*

*stable, you understand … I know that I am only with my husband, but you don't know if he is only with you …“* (FGD 4)

According to most of the women, since male infidelity was present in many couples, women should be very alert and perceive if their partners have STD in order to protect themselves. It was also mentioned that women should avoid sexual contact with persons they do not know as a way of protection against STDs.

*“Women should know with who they maintain relations and they should know*

*the symptoms of each disease that people can have, that is they have to be conscious about gonorrhoea, syphilis, papilloma and all those things, I imagine they should know to protect themselves*”(FGD 10)

In one of the FGD, it was discussed that there were more health problems related to STDs in Brazil than in Venezuela because promiscuity was more common in Brazil than in their country.

*“I think that here in Brazil females have more liberty and that is the cause … in*

*Venezuela homosexuality is not eradicated, it exists but it is not legalized … promiscuity with some modesty … for example it's not so open, independently of the situation there being much more difficult than here, you can't get condoms, and people are more careful. HIV is not so common as here*.” (FGD 6)

In two other FGDs it was mentioned that there had been some educational activities in the shelters on HIV/STDs. In one of those groups the need of campaigns to perform exams with the people who were arriving at the shelters to detect if they had some STD was discussed. Moreover, it was considered important for the adoption of means for prevention. Mixed with this discussion participants mentioned that there were women, men and LGBTQI+ people living in the shelter who were sex workers in the city and considered that these people could be contaminated or even have STDs and were putting at risk the health of other people living in the shelter.

*”I think that because of those things they should have educational activities for*

*all the people in the shelter; everybody should do exams and start to work with these issues, because it is very delicate. Here we have many women that work as prostitutes at the central part of the city … if they use the necessary protection no avoid getting these diseases. They use the same bathrooms as we the women who do not leave the shelter … we don't know who is infected. This is not good for the shelter …*” (FGD 9)

## Discussion

To the best of our knowledge, this is the first qualitative report that presents perspectives and views on SRH issues and services of Venezuelan migrant women at reproductive age hosted at shelters established by the UNHCR at the north-western border between Brazil and Venezuela. Our results, based on data collected through FGDs in 2020, referred to the perspectives and views of women relatively newly arrived at the shelters were they were hosted for a transitory period of time before the resettlement to different regions of Brazil.

The resolution of SRH needs is an important issue among these migrant women including the preoccupation with access and the understanding of the functioning of the Brazilian healthcare services. These preoccupations are important, mainly among pregnant women who were worried about giving birth, postnatal care and contraception and similar to the concerns observed among Venezuelan migrant women in Colombia (14; Rivillas-García et al., 2021).

In our study, the FG participants’ were in general satisfied with the health attention they received at the Maternity hospital. However, they were surprised about the rules of the healthcare professionals at this facility in favour of vaginal rather than Caesarean delivery, since their expectations were Caesarean delivery to avoid suffering. This is a frequent expectation among women in several contexts and may be related to specific cultural factors, fear of pain during childbirth, previous experience and interactions with healthcare professionals (O´Donovan and O´Donovan, 2018). The Maternity hospital in Boa Vista is the only public hospital dedicated to Obstetrics and Gynaecology and this facility is overwhelmed. The proportion of deliveries with the arrival of the Venezuelan migration increased from 3.4% in 2016 to 23.2% during 2019 (Brazil; Nascer no Brasil). Furthermore, the Caesarean delivery rate at that hospital in the year 2019 was 35.9% lower than the 46% in the Brazilian public sector network (Nascer no Brasil). Brazil has invested in the last 20 years in improving the Obstetric attention at the public health system through training of the healthcare providers, implementing and increasing counselling at antenatal, childbirth and postnatal care, to avoid obstetric mistreatment and to reduce the high rates of Caesarean deliveries in the country (Brasília. Ministério da Saúde 2017; OMS. Salud Materna 2016), considering that Brazil is the Latin American country with the highest rate of Caesarean deliveries (26).

Despite the general satisfaction with obstetric care, women noted few challenges pertaining to their experiences during labour and delivery. We can speculate that language was one of the main barriers as women were not able to fully understand the information given to them both at antenatal or postnatal care and particularly during labour and childbirth, as previously reported regarding migrant women´s perspective on antenatal and postpartum care (14; Miller et al., 2016; Santiago and Figueiredo, 2015). Concomitantly, during FGDs it was referred that women in labour and delivery were pleasantly surprised and liked that they were allowed to have a companion of their choice to be with them during labour, which they noted to be uncommon in Venezuela. This procedure of the right of companionship during labour was institutionalised in Brazil based on the norms established for a more humanised obstetric attention of the Ministry of Health (Brasília. Ministério da Saúde 2017).

In general, satisfaction of the women during the birthing process is related with the support they received and benefits of this support for women and infants have been described (Hodnett et al., 2013). This is in line with the WHO recommendations that highlighted the importance of birthing in an adequate sanitary environment and of humanised attitudes during attention. This guideline based on a holistic, human rights-based approach reinforces the importance of the woman-centred care to facilitate a satisfactory environment for labour and childbirth. This human right is particularly important for those in critical situations like migrant women (OMS. Salud Materna 2016).

The women attributed the large number of Venezuelan pregnant women at the shelters because some women migrated already pregnant, seeking adequate and free of charge health services that were not available in Venezuela. Although in Venezuela the health care services are free of charge at the public sector, due to the crisis, women are required to bring many medicine and materials for their treatment at the time of admission to the hospitals (14). In Brazil health care attention is considered a human right for all the citizens (nationals, residents or migrants) and all the attentions at the public services are at no cost for patients. The National Health Service (*Sistema Único de Saúde*, SUS) has been the main source for health care of 75% of all citizens (Campos, 2018; Kleinert and Horton, 2011). This free of charge access to health attentions for all including non-legal documenting migrant is an advantage different than in other hosting countries were health care services are not always free of charge and in many situation exclude migrant women seeking SRH services (14).

Furthermore, participants considered that some women became pregnant purposefully because they know that in Brazil, the law established that parents of children under 18 years old who are born in the country have the right to obtain legal residence (Brasil). Further, abortion did not emerge as a theme during the FGDs, and this finding is surprising given that previous data refers to an estimated 17% of the Venezuelan migrant women in Brazil who reported a history of at least one abortion before migration (14). It could be possible that women in the FGDs refrained from talking about abortion because they were aware that the Brazilian law is restricted about medical termination (Theme-Filha et al., 2016), different from Colombia were policies tend to be more permissive (14).

Contraception both at the postpartum and interval periods was also discussed during the FGDs. Access to contraceptives for all women including Venezuelan migrant women is free of charge in Brazil. The women were in agreement that access to some short-acting contraceptives was less difficult than to long-acting reversible contraceptives (LARCs), mainly to the Cu-IUD, when available access was erratic. Lack of access to contraceptives was a major concern to the women; however, is not only prevalent among Venezuelan migrant women; but is also a common problem among women living in Brazil. The overall prevalence of modern contraceptive methods in Brazil is almost 75% (United NationFund for Population Affaires (UNFPA)); however, the rate of unplanned pregnancies is high (52%) (Theme-Filha et al., 2016) in part as a consequence of the lack of access to LARCs methods. Although Cu-IUD is available at all public health facilities at no cost there are a scarce number of providers trained in placement and management (Gomez Ponce de Leon et al., 2019).

Contraceptive implants and hormonal-IUD are almost not available in the Brazilian public health network, posing a difficulty of access to these methods not only for Venezuelan but also for Brazilian women. In the context of migration, access to LARCs is important because these methods present high contraceptive efficacy (Winner et al., 2012) which is especially important for migrants while they strive towards relatively stable life circumstances. Our results showed a more equitable situation for Venezuelan migrant women regarding access to contraceptives when compared to the situation of Venezuelan women who migrated to Colombia were they have to pay for these services, and have restricted access to contraceptives. Consequently, Venezuelan women presented low coverage and inequitable access when compared to the host population (Rivillas-García et al., 2021).

Women’ knowledge on STDs/HIV prevention and transmission was adequate; a fair proportion of the FGDs participants knew the main risk factors for sexual transmission of these diseases and agreed that the use of condom and monogamy are the two effective means for prevention. The predominance of traditional gender imbalance in the relations was observed among the participants, a similar situation was identified among Latino migrant women in the USA (Daniel-Ulloa et al., 2016). Also, these attitudes have been discussed as a barrier for migrant women, in general, to protect themselves against HIV/STD infection (Webber, 2007; Gupta et al., 2008).

The fact that participants considered that extramarital relationships as inevitable for men evidenced an interaction of views regarding gender, sexuality, social roles and power. This fact necessitates a comprehensive and structural approach to reduce women's vulnerability to HIV/STDs infections and it equally reflects a gender imbalance among women (Gupta et al., 2008; Kippax et al., 2013). It is important to take into account these women's views on HIV/STD prevention and risks in order to organise assertive and effective interventions with the power of transformative actions, addressing inequalities and offering meaningful information. Brazil has a pioneering role in the prevention of HIV that led to the development of progressive and effective policies (41).

Our study presents strengths. To the best of our knowledge, this is the first report that sheds the light on Venezuelan migrant women's SRH issues, concerns and challenges at the Brazilian border. This study allowed women to discuss their SRH needs and perspectives as well as present views they have on SRH. Our study also presents limitations. The FGDs were limited to one of the Brazilian region in which migration occurs (although it was the largest point of entry for these migrants), we did not interview adolescents under 18 years old and we did not interview migrant women who are living outside shelters.

## Conclusion

Our results describe the needs of Venezuelan migrant women as perceived by them during the first phase of migration to Brazil. Based on their perceived needs, it can be concluded that these women need help to understand the language, the organisation and functioning of the healthcare system, since they continue to strive for the same health care access as that they used in their own country.

We believes that our findings can be informative for national and local health authorities, as well as, international organisations, in order to drive a more focused- evidence informed response that cater to the specific SRH needs and challenges of this migration crisis. For example, some important response strategies could include health awareness and capacity building around the essential SRH services for migrants, healthcare providers, policy makers and stakeholders. This could allow for improving the quality of health care in the existent facilities as well as to better respond to the needs of these women. Our findings provide some light on areas for additional training needs for staff both at shelters and at local health facilities, to help with the understanding of the needs migrant women have, as well as, their perspectives and views; and to develop more adequate approaches and strategies for dissemination of information on SRH issues and care for migrant women (Schmidt et al., 2018).

### Highlights

We like to highlight that our findings are important in the present time in which the world faces serious fragility in the healthcare network due to the SARS-CoV-2 pandemic, which in many cases closed and rendered many SRH services, as result of the reallocation of many healthcare providers to attend COVID-19 patients (Bahamondes and Makuch, 2020). Similar situation was recently described in Colombia and Peru in which migrants discovered obstacles to reach the healthcare system like legal, financial and discrimination which impairs the migrants’ ability to cover the costs of basic needs and worsened health inequities of Venezuelan migrants (Zambrano-Barragán et al., 2021). We also would like to highlight that maintaining essential SRH services is a priority in the framework of universal health coverage, and especially so at the times of this pandemic, when SRH needs of the community and migrants are likely to become more exposed and challenged (41; Schmidt et al., 2018; Bahamondes and Makuch, 2020). Hence, at the moment, this matter is of great concern.

## Declaration of Competing Interest

The authors have declared that no competing interests exist.

## Funding

Preparatory work for this study was partially funded by the World Health Organisation (WHO). The research protocol was prepared by the study research group and does not necessarily reflect the views of the funding partners. This article represents the views of the named authors only.

## CRediT authorship contribution statement

**Maria Y. Makuch:** Conceptualization, Formal analysis, Supervision, Validation, Writing – original draft, Writing – review & editing. **Maria Jose D. Osis:** Conceptualization, Formal analysis, Supervision, Validation, Writing – original draft, Writing – review & editing. **Cinthia Brasil:** Writing – original draft, Writing – review & editing. **Helder S.F. de Amorim:** Writing – original draft, Writing – review & editing. **Luis Bahamondes:** Conceptualization, Formal analysis, Funding acquisition, Project administration, Supervision, Validation, Writing – original draft, Writing – review & editing.
